# Novel Approach to Elucidate Human Baroreflex Regulation at the Brainstem Level: Pharmacological Testing During fMRI

**DOI:** 10.3389/fnins.2019.00193

**Published:** 2019-03-05

**Authors:** Darius A. Gerlach, Jorge Manuel, Alex Hoff, Hendrik Kronsbein, Fabian Hoffmann, Karsten Heusser, Heimo Ehmke, André Diedrich, Jens Jordan, Jens Tank, Florian Beissner

**Affiliations:** ^1^Department of Cardiovascular Aerospace Medicine, Institute of Aerospace Medicine, German Aerospace Center (DLR), Cologne, Germany; ^2^Somatosensory and Autonomic Therapy Research, Institute for Neuroradiology, Hannover Medical School, Hanover, Germany; ^3^Institute of Cellular and Integrative Physiology, University Medical Center Hamburg-Eppendorf, Hamburg, Germany; ^4^Division of Cardiology, Angiology and Pneumology, Cologne Heart Center, University Hospital Cologne, Cologne, Germany; ^5^Division of Clinical Pharmacology, Department of Medicine, Autonomic Dysfunction Service, Vanderbilt University, Nashville, TN, United States; ^6^Chair of Aerospace Medicine, Institute of Aerospace Medicine, German Aerospace Center (DLR), Helmholtz Association of German Research Centers, Cologne, Germany

**Keywords:** baroreflex, fMRI, brainstem, blood pressure, regulation, cardiovascular, nuclei

## Abstract

**Introduction:** Brainstem nuclei govern the arterial baroreflex, which is crucial for heart rate and blood pressure control. Yet, brainstem function is difficult to explore in living humans and is therefore mostly studied using animal models or postmortem human anatomy studies. We developed a methodology to identify brainstem nuclei involved in baroreflex cardiovascular control in humans by combining pharmacological baroreflex testing with functional magnetic resonance imaging.

**Materials and Methods:** In 11 healthy men, we applied eight repeated intravenous phenylephrine bolus doses of 25 and 75 μg followed by a saline flush using a remote-controlled injector during multiband functional magnetic resonance imaging (fMRI) acquisition of the whole brain including the brainstem. Continuous finger arterial blood pressure, respiration, and electrocardiogram (ECG) were monitored. fMRI data were preprocessed with a brainstem-specific pipeline and analyzed with a general linear model (GLM) to identify brainstem nuclei involved in central integration of the baroreceptor input.

**Results:** Phenylephrine elicited a pressor response followed by a baroreflex-mediated lengthening of the RR interval (25 μg: 197 ± 15 ms; 75 μg: 221 ± 33 ms). By combining fMRI responses during both phenylephrine doses, we identified significant signal changes in the nucleus tractus solitarii (*t* = 5.97), caudal ventrolateral medulla (*t* = 4.59), rostral ventrolateral medulla (*t* = 7.11), nucleus ambiguus (*t* = 5.6), nucleus raphe obscurus (*t* = 6.45), and several other brainstem nuclei [*p* < 0.0005 family-wise error (few)-corr.].

**Conclusion:** Pharmacological baroreflex testing during fMRI allows characterizing central baroreflex regulation at the level of the brainstem in humans. Baroreflex-mediated activation and deactivation patterns are consistent with previous investigations in animal models. The methodology has the potential to elucidate human physiology and mechanisms of autonomic cardiovascular disease.

## Introduction

Cardiovascular control centers in the brainstem govern arterial baroreflexes, which are important for human blood pressure buffering (Jordan et al., 2002) and long-term blood pressure control (Bisognano et al., 2011). Careful physiological investigations in animals showed that the NTS is the primary relay station for afferent input from carotid and aortic baroreceptors (McAllen and Spyer, 1976; Dampney, 1994). Connections from there to the NA and DMN elicit counter regulatory adjustment in efferent cardiac vagal activity. Projections to the cVLM and from there to the rVLM adjust efferent sympathetic traffic (Dampney, 1994; Dampney et al., 2002). Damage to these brainstem nuclei results in profound abnormalities in human blood pressure control. A patient with ischemic lesions involving bilateral NTS featured afferent baroreflex failure (Biaggioni et al., 1994). Degeneration of brainstem nuclei including the rVLM in patients with multiple system atrophy is associated with severe orthostatic hypotension among other disabling symptoms of efferent baroreflex dysfunction (Benarroch et al., 1998). Even subtle abnormalities in the structure or function of these nuclei could substantially affect human cardiovascular regulation. Yet, while the overall integrity of arterial baroreflex function can be interrogated with physiological and pharmacological baroreflex tests (Bristow et al., 1971, 1974), baroreflex regulation at the level of the brainstem is very difficult to measure in humans. Our goal was to develop a novel approach to assess human baroreflex regulation at the level of the brainstem. Therefore, we combined fMRI of the BOLD, beat-by-beat blood pressure and heart rate monitoring, and phenylephrine bolus injections for pharmacological baroreflex loading. Phenylephrine increases blood pressure leading to baroreflex-mediated vagal activation and sympathoinhibition. Brainstem fMRI has previously been validated for several applications including trigeminal pain research (Schulte et al., 2016), resting state connectivity measurement (Beissner et al., 2014), characterization of the autonomic nervous system (Macefield and Henderson, 2010; Coulson et al., 2015), and studies on sleep apnea (Henderson et al., 2016).

## Materials and Methods

### Subjects

We included 11 healthy, normotensive (125.7 ± 4.6/73.4 ± 5.5 mmHg, 57.6 ± 7.5 bpm), non-smoking, men aged 30.5 ± 6.3 years with a weight of 78.0 ± 10.6 kg ranging from 65 to 98 kg and a body mass index of 24.0 ± 1.9 kg/m^2^. Subjects were normally active and non-sedentary. The study complied with the Declaration of Helsinki was approved by the ethics committee of the Ärztekammer Nordrhein, Düsseldorf, Germany, and all subjects had given their written informed consent before inclusion. We registered the study under clinical trials registration number DRKS00013101 prior to commencement.

### Study Design

The study was a randomized controlled interventional trial in an ambulatory setting. Subjects visited the facility on three different days. On the first study day, subjects were familiarized with the MRI environment and physiological recording equipment and underwent medical examination. On the second study day, the subject’s baroreflex response to phenylephrine boli was examined. Finally, pharmacological baroreflex testing during fMRI took place on the third study day. Subjects rested before the scan and had abstained from caffeine and alcohol for 24 h. After positioning and instrumentation of the subject followed by a resting period of 20 min in the scanner, we applied repeated intravenous phenylephrine (25 and 75 μg, *n* = 8) boli followed by a 10 ml normal saline flush using a programmable, MR-compatible remote-controlled injector. We repeated bolus administration every 120 s with *n* = 8 boli in total. Each fMRI run lasted 16 min 49 s. The 25 and 75 μg doses were applied in separate runs in randomized order. MRI measurements were carried out between 8:40 a.m. to 12:05 p.m. in an air conditioned room kept at a constant 21°C. All subjects were asked about their mood, sleep, and mental state using a customized non-standardized questionnaire before and after the examination. In particular, we asked about sleepiness, pain, and whether the subject perceived any effects of the injection.

### MRI Acquisition

We obtained MRI acquisitions with a 3 T scanner (mMR Biograph PET-MRI scanner based on the Verio system, Siemens, Erlangen, Germany) with a 32-channel head coil. T1-weighted images for anatomical references were acquired using a MPRAGE sequence with the following parameters: TR: 2400 ms, TE: 2.13 ms, TI: 1000 ms, flip angle: 8°, FOV: 246 mm∗192 mm, matrix: 246∗192, slice thickness: 1 mm. T2^∗^-weighted functional images were acquired with an EPI sequence accelerated by multiband acquisition (TR/TE: 1180/32 ms, flip angle: 64°, FOV: 180 mm∗208 mm, matrix: 90∗104, slices: 78 with 2.0 mm slice thickness, voxel size: 2.0 mm isotropic, multiband factor: 6, volumes: 846; Xu et al., 2013; Todd et al., 2016). Unaccelerated single EPI images (TR: 6127 ms, flip angle: 90°) and B0-weighted spin echo EPI (TR/TE: 12,000/102.6 ms) with matched and 180° rotated phase encoding direction were acquired for better gray-white contrast and distortion correction, respectively. The flip angle was chosen according to the Ernst angle for shortened TR. Scans covered the whole brain including the brainstem. The total scan time was 45–50 min per intervention and subject. Image orientation was parallel to the anterior-posterior commissure line for T1-weighted images, whereas functional MRI images were additionally tilted by ∼35° to avoid signal drop-outs in areas of interest. The MRI protocol was optimized according to the findings and recommendations from the human connectome project (Ugurbil et al., 2013). Additional information on MR imaging sequences for the non-specialist can be found in Bitar et al. (2006).

### Physiological Recordings

We recorded beat-to-beat finger blood pressure with a modified device based on a commercially available finger blood pressure monitor (NOVA^®^, FMS, Finapres Measurement Systems, Amsterdam, Netherlands). For MRI compatibility, the device was radio frequency shielded and the cuff to frontend distance was prolonged to increase the frontend’s distance to the scanner. We also acquired the ECG and SpO_2_ (MR400, PHILIPS, Orlando, FL, United States) as well as respiratory rate and end-tidal CO_2_ (etCO_2_; IVY 450C, Branford, CT, United States). Signals were collected after A/D conversion (WINDAQ, DATAQ, Akron, OH, United States) with a sampling frequency of 500 Hz. Data preprocessing included peak detection, outlier filtering, resampling to fMRI acquisition, and normalization. RR intervals, systolic and diastolic blood pressure values, and respiration rate for each heartbeat and breathing cycle were analyzed. Subjects were equipped with active noise canceling headphones during the fMRI scan (OptoACTIVE, Optoacustics Ltd., Mazor, Israel). A fixed head position was maintained by inflatable pads around the headphones.

### Image Analysis and Statistics

We preprocessed fMRI data with FSL tools, v5.0.11 (Oxford Centre for Functional MRI of the Brain, Oxford, United Kingdom; Smith et al., 2004; Woolrich et al., 2009). After conversion of fMRI images to NIFTI format, all multiband EPI were realigned to the unaccelerated EPI image using FSL MCFLIRT. This approach allows for motion correction. At the same time, unwarping was conducted with FSL TOPUP using the spin-echo EPI for distortion correction (Andersson et al., 2003). Multiband EPI were then brain extracted with FSL BET, high-pass filtered with 180 s cut-off, and spatially normalized to a study template made from the T1 and unaccelerated EPI images of all subjects. Template and transformation for registration were calculated using ANTs (Tustison and Avants, 2013). The final preprocessing step was the upsampling to the T1 study template with ANTs.

The preprocessed data were cropped to retain only the lower brainstem and a brainstem mask was applied to remove adjacent areas with high physiological noise (Beissner et al., 2014). However, no spatial smoothing was applied in our study.

**FIGURE 1:**
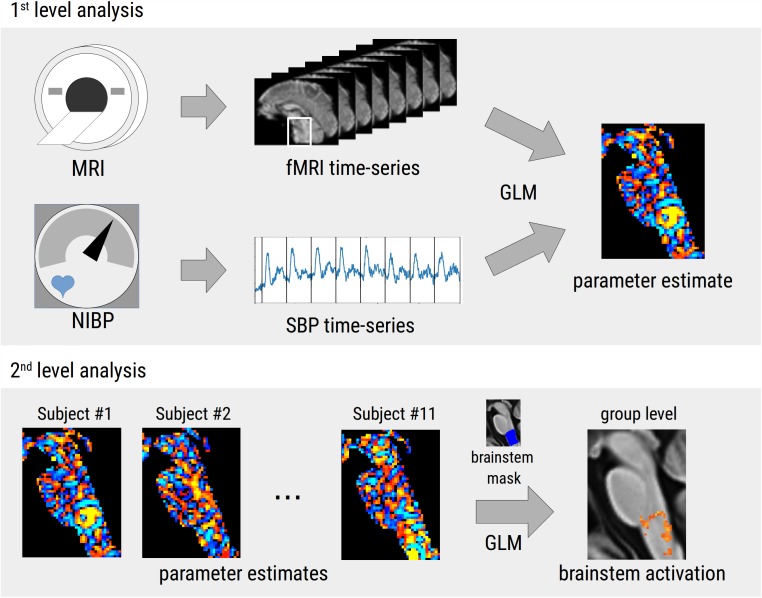
Flow chart of the two-step statistical approach used in the study. In the first-level analysis, the time-course of systolic blood pressure (SBP) recorded during repeated phenylephrine bolus injections was used as regressor in a general linear model (GLM) on the functional MRI time-series leading to voxel-wise parameter estimate maps. In the second-level analysis, parameter estimates of all subjects were combined in a second GLM to calculate a group level statistical parametric map that was thresholded using family-wise error correction and threshold-free cluster enhancement. All analyses were restricted to the lower brainstem.

Statistical analysis was done with a mixed-effect GLM (Figure 1). The full SBP time-course of 16.5 min was used as explanatory variable and regressed against the BOLD signal time-courses of individual voxels. First-level (single-subject) analyses were performed with FSL_GLM and the parameter estimates passed up to a second-level (group) analysis using non-parametric permutation testing with FSL RANDOMISE. Significance was assumed at *p* < 0.0005 corrected for multiple comparisons using family-wise error (FWE) correction and threshold-free cluster enhancement (Smith and Nichols, 2009). Results were reported in the form of *t*-values (defined as the parameter estimate from the GLM divided by the error of the parameter estimate). After statistical analysis, results were transformed to standard space (Montreal Neurological Institute, MNI152 1 mm brain) using ANTs. We further analyzed the correlation between the averaged BOLD time-courses with the group mean SBP by extracting the BOLD signal from the masked lower brainstem.

## Results

### Pharmacological Baroreflex Testing

**FIGURE 2:**
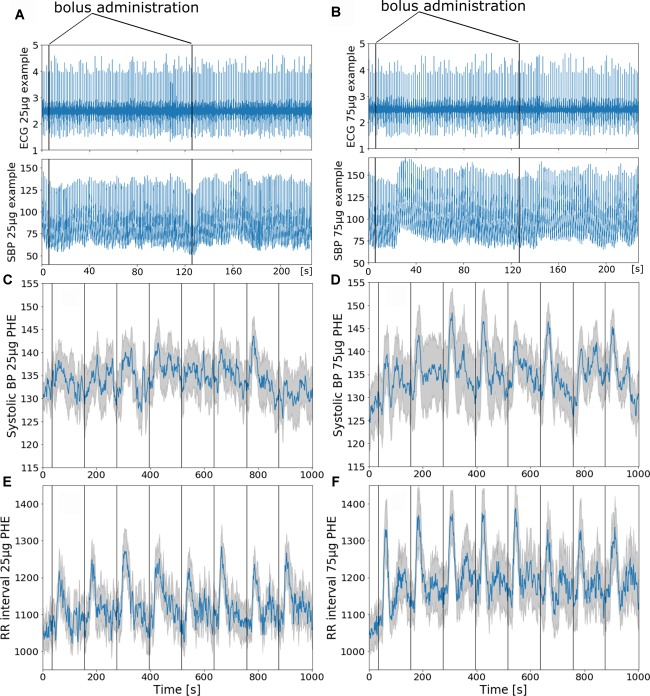
**(A,B)** Phenylephrine bolus administration (**A**: 25 μg and **B**: 75 μg) with blood pressure and ECG recordings during functional MRI. The vertical black lines indicate bolus injections. **(C–F)** Group mean ± SE (gray curve) physiological recordings from 11 subjects with repeated phenylephrine administration. Systolic pressure **(C)** and RRI from ECG **(E)** recordings during 25 μg phenylephrine and systolic pressure **(D)** and RRI **(F)** recordings during 75 μg phenylephrine bolus administration.

All subjects reported to have slept well the night before the testing and were well motivated. None of the subjects reported pain provoked by the injection or by lying in the scanner, and all managed to stay awake throughout the test. We obtained good quality finger blood pressure recordings in most instances over the imaging period in 10 out of 11 subjects; in one subject, the signal drop outs were interpolated. Blood pressure traces deteriorated toward the end of the experiment in six of 11 subjects. To compensate for MRI induced artificial signal declines the regularly repeated level – and gain – calibration option (Physiocal^TM^) was used in some subjects when needed. This resulted in a signal dropout of few seconds that was linearly interpolated. Still, all subjects could be considered for further analysis. Figure 2A,B illustrate continuous ECG and finger blood pressure recordings during administration of a 25 μg and a 75 μg phenylephrine bolus dose. Figure 2C–F illustrate averaged finger blood pressure RR interval responses to repeated phenylephrine doses; 25 and 75 μg doses increased SBP 5 ± 2 and 15 ± 2 mmHg, respectively. The pressor response elicited baroreflex-mediated heart rate reductions (RR interval lengthening 197 ± 15 ms with 25 μg: and 221 ± 33 ms with 75 μg). Despite the relatively short period between the repeated boli of only 2 min blood pressure did not increase over the 16.5 min imaging time. Baroreflex sensitivity estimation resulted in mean values13.8 ± 7.4 ms/mmHg for 25 μg and 14.1 ± 4.6 ms/mmHg for 75 μg ranging from 3.4 to 40.1 ms/mmHg. Baroreflex sensitivity was not always detectable especially during the 25 μg doses.

### Brainstem fMRI

The GLM of the BOLD signal with SBP revealed significant activations (i.e., positive correlation) on the group level. Thus, an increase in SBP was related to an increase of the BOLD signal in the respective voxels. The analysis was first conducted separately for 25 and 75 μg phenylephrine doses, resulting in a single significant voxel for the 75 μg runs that was located in the NTS. A paired *t*-test between the 25 and 75 μg bolus administrations showed no differences, which led us to pool the data and improve statistical power. Based on this pooled analysis, we found significant activations in a number of brainstem nuclei that were subsequently identified using the Paxinos brainstem atlas (Paxinos et al., 2012). These nuclei comprised the NTS, cVLM and rVLM, ROb, DMN, nucleus hypoglossus (12N), inferior olive (IO), and different reticular nuclei. MNI coordinates and corresponding *t*-values are shown in Table 1.

**Table 1:** Identified brainstem nuclei.

Side	*t*-value	MNI: *x* (mm)	MNI: *y* (mm)	MNI: *z* (mm)	Brainstem nuclei
r	7.21	5	-38	-60	Inferior olive (IO)
l	7.11	-8	-38	-46	Rostral ventrolateral medulla (rVLM)
					Lateral reticular nucleus (LRt)
					Lateral paragigantocellular nucleus (LPGi)
l/r	6.62	2	-43	-51	Hypoglossal nucleus (12N)
	6.45	1	-45	-57	Raphe obscurus nucleus (ROb)
l	6.29	-3	-43	-56	Intermediate reticular nucleus (IRt)
l	5.97	-1	-45	-54	Dorsal motor nucleus of the vagal nerve (DMN)
					Nucleus tractus solitarii(NTS)
r	5.67	7	-34	-45	Rostral ventrolateral medulla (rVLM)
r	5.67	2	-40	-44	Dorsal paragigantocellular nucleus (DPGi)
r	5.61	7	-42	-56	Spinal trigeminal nucleus (SP5)
r	5.6	6	-40	-48	Nucleus ambiguus (NA)
l	4.98	-7	-44	-56	Spinal trigeminal nucleus (SP5)
r	4.59	6	-38	-55	Caudal ventrolateral medulla (cVLM)
l	4.72	-5	-46	-53	Spinal vestibular nucleus (SpVe)
l	4.43	-6	-35	-55	Inferior olive (IO)
r	4.4	6	-45	-50	Medial vestibular nucleus
l	4.05	-5	-42	-48	Nucleus tractus solitarii (NTS)

*All brainstem nuclei encompassed in the cluster were identified using a brainstem atlas. Active regions after p < 0.0005 threshold are reported in MNI standard space coordinates with the corresponding t-values for the local maxima. (Larger t-values indicate better correlation between fMRI signal and systolic blood pressure. The t-value is defined as the parameter estimate from the GLM divided by the error of the parameter estimate.) Cluster size: 1437 (mm^3^), center of gravity x: 1.46, y: -40, z: -51.6. MNI: Montreal Neurological Institute.*

**FIGURE 3:**
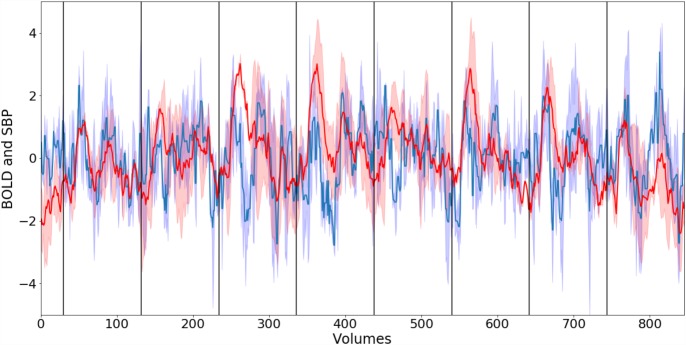
Normalized smoothed group mean BOLD signal (blue) from lower brainstem and group mean systolic blood pressure (SBP) (red). Corresponding blue and red areas depict standard error. Black vertical lines indicate the start of phenylephrine bolus injections. The correlation R between both time-courses is 0.25.

The relationship between the averaged time-courses of the whole lower brainstem BOLD signal and SBP is depicted in Figure 3. Both signals exhibit strong variations and a poor correlation (*R* = 0.25). Thus, blood pressure changes alone are most likely not the only contributor to the fluctuations of the BOLD signal.

**FIGURE 4:**
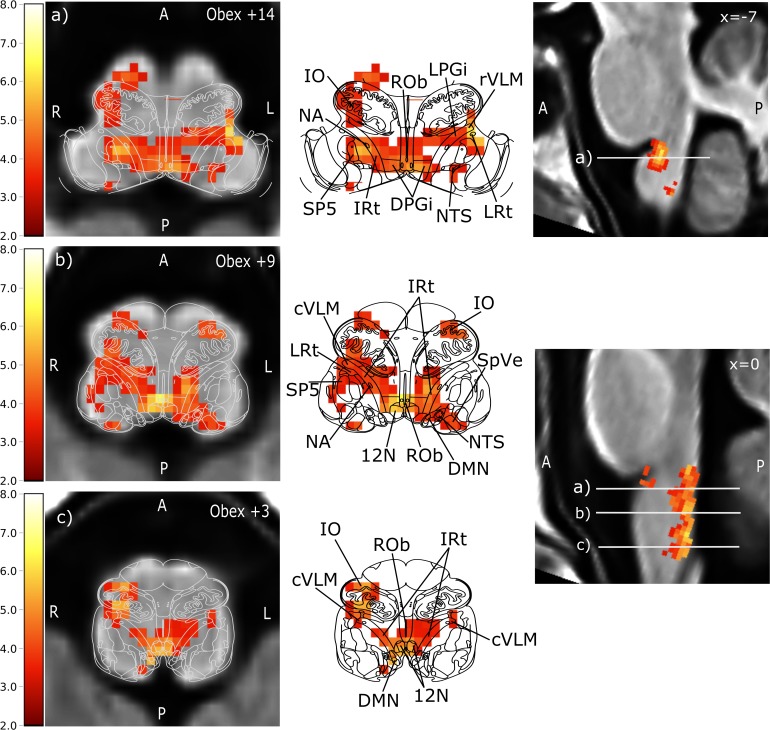
Brainstem regions showing activations associated with blood pressure changes elicited by phenylephrine bolus injections. Group level results of all 11 subjects using data from all 16 boli. For visualization purposes, the images were tilted such that the resulting sections were perpendicular to the rostro-caudal brainstem axis to match the anatomical atlas. Left: transversal lower brainstem slices with of anatomical group template overlaid with the statistical parametric map of the positive BOLD correlation with SBP (*t*-values encoded by color scale) and the corresponding atlas slice (modified from Paxinos brainstem atlas). Middle: BOLD overlay with the Paxinos brainstem atlas (Paxinos et al., 2012). Right: sagittal view of the brainstem. The corresponding transversal slices are marked by white lines with the letters of the sub-figure. Prominent activation maxima include **(a)** left rostral ventrolateral medulla (rVLM), Ncl. raphe obscurus (ROb), and right Ncl. ambiguus (NA), **(b)** Ncl. raphe obscurus (ROb), left intermediate reticular nucleus (IRt) extending to nucleus tractus solitarii, and right caudal ventrolateral medulla (cVLM), and **(c)** Ncl. hypoglossus (12N) extending to IRt and dorsal motor nucleus of the vagal nerve (DMN), right inferior olive extending to the cVLM. Further activated nuclei are: lateral reticular nucleus (LRt), dorsal paragigantocellular nucleus (DPGi), lateral paragigantocellular nucleus (LPGi), spinal trigeminal nucleus (SP5) and spinal vestibular nucleus (SpVe). A: anterior, P: posterior, L: left, R: right.

Figure 4 illustrates the significant group level activations. Overlaid anatomical atlas slices were used to identify the nuclei.

## Discussion

The important finding of our study is that pharmacological baroreflex testing combined with fMRI reveals brainstem nuclei involved in human baroreflex regulation. In humans, we identified with high sensitivity all of the brainstem nuclei that have previously been shown to contribute to the baroreflex circuit in animals. Our approach can now be applied to elucidate the role of the human brainstem in cardiovascular physiology and in the pathogenesis of human cardiovascular disease.

The combination of continuous cardiovascular monitoring and brainstem fMRI during baroreflex loading with phenylephrine is a particular strength of our study. Beat-by-beat blood pressure can so far only be assessed non-invasively with volume-clamp methods based on the Penàz servo-plethysmomanometer (Molhoek et al., 1984). Devices based on this principle (i.e., NOVA^®^, FMS, Finapres Measurement Systems, Amsterdam, Netherlands) are crucial for interrogating baroreflex function, need to be heavily modified for MRI studies and are only available in a few laboratories worldwide (Critchley et al., 2015). Other than the volume-clamp principle, there are commercially available non-invasive blood pressure systems for MRI (i.e., Biopac Systems Inc., Goleta, CA, United States) that are based on pulse decomposition analysis. The methodology is rather an indirect measure for the blood pressure (Baruch et al., 2011). Furthermore, brainstem fMRI is still challenging because of the strong physiological noise sources surrounding it (Beissner et al., 2014; Beissner, 2015). Due to the small size of brainstem nuclei, fMRI methods optimized for cortical structures cannot be applied (Beissner, 2015). Moreover, respiration, blood flow, and cerebrospinal fluid pulsations produce magnetic field distortions, out of phase spins and structural displacement (Brooks et al., 2013). Therefore, we aimed at maximizing statistical power by applying repeated stimulation with phenylephrine in a highly standardized fashion. To avoid non-specific effects of phenylephrine on brain circulation, we applied low and moderate phenylephrine doses (La Rovere et al., 1998). Baroreflex loading with phenylephrine produces an afferent signal that is conveyed to the NTS. Baroreflex afferent recordings in animal experiments and in patients during carotid surgery showed that signal time-course and magnitude were related to blood pressure. Since afferent nerve signals cannot be reasonably recorded in a human study, we utilized beat-by-beat blood pressure as input for our GLM analysis. We are aware that afferent baroreceptor input to NTS also feeds back on blood pressure. Compared with prior studies, our approach yields several advantages in delineating central baroreflex control. Previously applied autonomic challenges during fMRI include LBNP (Kimmerly et al., 2005), isometric handgrip testing (Coulson et al., 2015), Valsalva maneuver (Henderson et al., 2002), and slow breathing (Critchley et al., 2015). Handgrip and cold pressor testing engage central autonomic circuits through muscle afferents and pain fibers rather than baroreflex input. The Valsalva maneuver requires active participation likely confounding fMRI analysis and its effects are entangled with that of transient hypercapnia. The major challenge of LBNP is that it induces movement artifacts, when subjects are sucked down into the chamber. Moreover, the input stimulus for fMRI is commonly conceptualized as a boxcar time-course (on vs. off). Instead, we measured blood pressure time-course and magnitude during pharmacological baroreflex loading. It should be noted that BOLD contrast captures changes in neural activity but cannot readily differentiate neural inhibition and activation over time (Logothetis, 2008).

We reasoned that a methodology assessing brainstem baroreflex integration should recapitulate known baroreflex circuits. Indeed, baroreflex loading with phenylephrine yielded positive correlations between BOLD signals in the NTS and SBP. We also observed baroreflex-related BOLD signal changes in the NA which governs efferent cardiac vagal activity. Furthermore, baroreflex loading significantly changed BOLD signals in nuclei involved in sympathetic control including cVLM, rVLM, and raphe obscurus and provided more detailed coverage of baroreflex-regulated brainstem nuclei compared with previous fMRI studies in humans. Thus, fMRI resting state measurements combined with consecutively recorded muscle sympathetic nerve activity (Macefield and Henderson, 2010) suggested positive correlations between efferent sympathetic activity and the rVLM BOLD signal and negative correlations between sympathetic activity and NTS, cVLM BOLD signals. In another study, BOLD signals increased in broad regions including rVLM and decreased in cVLM and in medullary dorsomedial regions during inspiratory capacity apnea compared to relaxed breathing conditions (Macefield et al., 2006). Activation of higher cortical centers was reported in a baroreceptor unloading study with LBNP including insular frontoparietal cortex, and cerebellum (Kimmerly et al., 2005). Our localization of the rVLM, one of the central regions of baroreflex regulation, shows excellent correspondence with coordinates previously reported from a study correlating BOLD and blood pressure responses during hypoxia and normoxia and various breathing maneuvers (Critchley et al., 2015). Additionally, the IO, different reticular nuclei, 12N, and ROb were activated although they are traditionally not linked to blood pressure regulation. Even though some investigators suggested that these nuclei contribute to blood pressure control (Smith and Nathan, 1966; Miura and Reis, 1971), their role is not fully understood, yet. Because our methodology reliably identified previously known baroreflex-related brainstem nuclei, now human brain areas not previously accessible or not considered to relate to baroreflex activity can be interrogated.

### Limitations

The BOLD signal is altered with changes in cerebral blood volume and blood flow (Logothetis, 2008). We cannot fully exclude that phenylephrine indirectly or directly affected cerebral circulation, which could affect fMRI analysis. In rabbits, phenylephrine increased blood pressure and cerebral blood flow while cerebral blood volume and de- and oxyhemoglobin were left unchanged (Koyama et al., 1990). The finding is reassuring since the latter influence the BOLD signal. Moreover, phenylephrine did not produce cerebral vasoconstriction in patients during anesthesia whereas the volatile anesthetic isoflurane increased cerebral blood flow (Strebel et al., 1998). In contrast, cerebral tissue oxygenation decreased with phenylephrine during anesthesia patients with unchanged cerebrovascular volume (Meng et al., 2012). However, none of these responses could explain specific BOLD signal changes in brainstem baroreflex circuits. Indeed, phenylephrine was also applied to elucidate baroreflex-mediated BOLD signal responses in cats before and after baroreceptor denervation (Henderson et al., 2004).

The number of subjects in our study is relatively low in comparison to conventional cortical fMRI studies, even though we were able to demonstrate significant activations based on the high number of stimuli used. Thus, our sample may not represent the average population, which should be considered when generalizing our results. However, most of our results are consistent with previous studies applying LBNP, Valsalva maneuver, hand grip, or inspiratory load (Macey et al., 2015). Finally, our spatial resolution of 2 mm isotropic limits our ability to clearly separate nuclei that are in close vicinity. For example, we cannot rule out that the activation of the DMN observed in our study was not based on signal spreading from the neighboring NTS. Future technological developments may mitigate this problem as may improved experimental designs.

### Perspectives

We developed a novel approach to elucidate human baroreflex regulation at the level of the brainstem. The methodology can be applied to investigate human physiology. Indeed, much of our knowledge on central nervous baroreflex integration relies on animal studies and it has been difficult translating these findings to human subjects. Furthermore, the methodology can now be applied to investigate conditions associated with altered baroreflex function, dissect out the localization of the dysfunction, and, perhaps, target treatments in a more rational fashion. For example, fMRI-based baroreflex testing could be utilized to differentiate rare central and peripheral autonomic failure syndromes at an earlier stage. In common cardiovascular disorders such as heart failure, impaired baroreflex function heralds a poor prognosis (La Rovere et al., 1998, 2013). Better mechanistic understanding may beget new treatment approaches. Finally, device-based therapies targeting baroreflex afferents through electrical carotid sinus stimulation have been recently developed and tested in patients with resistant arterial hypertension and with heart failure (Heusser et al., 2010; Scheffers et al., 2010; Gronda et al., 2014). However, the response to electrical carotid sinus stimulation is variable and the proportion of non-responders is unacceptably high. Perhaps, brainstem studies could be developed further and then utilized to identify patients that are more or less likely to respond. To achieve these goals, the methodology should be tested in more detail, particularly in conditions associated with baroreflex impairment. Furthermore, the imaging methodology should be further refined. For example, more sophisticated analyses like masked independent component analysis (Moher Alsady et al., 2016) and frequency-based analysis of BOLD signals in resting-state settings (Chang and Glover, 2010) could prove useful.

## Data Availability

The raw data supporting the conclusions of this manuscript will be made available by the authors, without undue reservation, to any qualified researcher. The datasets generated for this study are available on request to the corresponding author.

## Author Contributions

DG was the study coordinator and contributed to fMRI physiological data acquisition and analysis and writing of the manuscript. JM contributed to scripting of preprocessing, discussion of analysis and results, and revision of the manuscript. AH contributed to adjustment of fMRI compatible setup and physiological data acquisition. HK contributed to subject recruitment and physiological data acquisition. FH contributed to subject supervision and physiological data acquisition. KH contributed to data review and interpretation and revision of the manuscript. HE revised the manuscript. AD contributed to physiological data analysis. JJ contributed to data discussion and revision of the manuscript and was the supervisor. JT contributed to study idea and revision of the manuscript and was the project supervisor. FB contributed to study idea, statistical analysis, discussion of analysis and results, and revision of the manuscript.

## Conflict of Interest Statement

The authors declare that the research was conducted in the absence of any commercial or financial relationships that could be construed as a potential conflict of interest.

## Abbreviations

12N: hypoglossal nucleus
ANTs: advanced normalization tools
BOLD: blood oxygenation level dependent contrast
cVLM: caudal ventrolateral medulla
DMN: dorsal motor nucleus of the vagus nerve
DPGi: dorsal paragigantocellular nucleus
ECG: electrocardiogram
EPI: echo planar imaging
FOV: field of view
fMRI: functional magnetic resonance imaging
FSL: FMRIB Software Library
FWR-corr.: family-wise error correction
GLM: general linear model
IO: inferior olivary nucleus
IRt: intermediate reticular nucleus
LBNP: lower body negative pressure
LPGi: lateral paragigantocellular nucleus
LRt: lateral reticular nucleus
MNI: Montreal Neurological Institute standard space
MPRAGE: 3D magnetization prepared rapid acquisition gradient echo
MRI: magnetic resonance imaging
NA: nucleus ambiguus
NTS: nucleus tractus solitarii
ROb: nucleus raphe obscurus
rVLM: rostral ventrolateral medulla
SBP: systolic blood pressure
SP5: spinal trigeminal nucleus
SpO2: oxygen saturation
SpVe: spinal vestibular nucleus
TE: echo time
TI: inversion time
TR: repetition time
